# Brief Report: Impact of Maintenance Pemetrexed Cessation on Clinical Outcomes of Patients With Metastatic Nonsquamous NSCLC

**DOI:** 10.1016/j.jtocrr.2024.100717

**Published:** 2024-08-24

**Authors:** Shreya Bhatia, Matthew Lu, Spencer Lessans, Michael Libre, Heidi Chen, Wade T. Iams

**Affiliations:** a Vanderbilt University School of Medicine, Nashville, Tennessee; b Department of Medicine, Division of Hematology-Oncology, Vanderbilt University Medical Center, Nashville, Tennessee; c Vanderbilt-Ingram Cancer Center, Nashville, Tennessee

**Keywords:** Non–small cell lung cancer, Chemoimmunotherapy, Pemetrexed, Pembrolizumab

## Abstract

**Introduction:**

Combination chemoimmunotherapy including pemetrexed and a PD(L)1 inhibitor is a common first-line systemic therapy approach for patients with metastatic nonsquamous NSCLC. Patients often discontinue maintenance pemetrexed due to adverse effects, and little is known about the impact of maintenance pemetrexed cessation on real-world progression-free survival (rwPFS) and overall survival (OS).

**Methods:**

A total of 121 patients with stage IV or recurrent, metastatic nonsquamous NSCLC treated at Vanderbilt University Medical Center (VUMC) were included in this retrospective analysis. Patients diagnosed between July 2017 and September 2023 were included if they received maintenance pemetrexed and pembrolizumab. Patients were divided into two groups: those who stopped pemetrexed due to toxicity and those who did not. rwPFS and OS were measured from time of stage IV or metastatic diagnosis to the date of radiographic progression or death, respectively.

**Results:**

Among patients with stage IV or recurrent, metastatic NSCLC (n = 121), who remained on maintenance pemetrexed and pembrolizumab (n = 68), the median rwPFS was 11.7 months (95% confidence interval [CI]: 7.47–not applicable [NA]) compared with 24.3 months (95% CI, 19.37–NA) among patients who stopped maintenance pemetrexed (n = 53) (*p* = 0.1). The median OS in the same patient groups was 25.8 months (95% CI: 13.8–NA) compared with 36.4 months (95% CI: 26.9–NA) (*p* = 0.15), respectively.

**Conclusions:**

In this study of patients with metastatic nonsquamous NSCLC who received maintenance pemetrexed and pembrolizumab, patients who stopped pemetrexed due to toxicity experienced similar outcomes to those who continued with pemetrexed. The optimal duration of maintenance chemotherapy should be further evaluated in the immunotherapy era.

## Introduction

Immune checkpoint inhibitors including a PD(L)1 inhibitor are now the frontline standard of care for most patients with metastatic NSCLC.^1^^,^^2^ Several common treatment regimens for patients with nonsquamous NSCLC combine a PD(L)1 inhibitor with maintenance pemetrexed for up to 2 years after completion of 4 cycles of induction platinum doublet plus PD(L)1 inhibitor.^3^, ^4^, ^5^, ^6^

Nevertheless, many patients encounter prohibitive toxicity during their maintenance pemetrexed course, and it is unknown whether stopping maintenance pemetrexed affects progression-free survival (PFS) or overall survival (OS). Herein, we explore the impact of maintenance pemetrexed cessation on real-world PFS (rwPFS) and OS in patients with metastatic nonsquamous NSCLC who received maintenance pemetrexed and pembrolizumab.

## Methods

### Study Design and Patient Population

A total of 214 patients with a confirmed diagnosis of metastatic (stage IV at initial diagnosis or recurrent, metastatic disease) NSCLC seen at Vanderbilt University Medical Center (VUMC) were identified through chart screening. Medical records were reviewed for validation and additional demographic and clinical information under IRB-approved protocol #131733. Patients with squamous cell carcinoma and patients who were never placed on maintenance pemetrexed and pembrolizumab were excluded. A total of 121 patients with stage IV or recurrent, metastatic nonsquamous NSCLC treated at VUMC between July 2017 and September 2023 were ultimately included in this retrospective analysis. Patients were then divided into the following 2 groups: those who stopped pemetrexed due to toxicity and those who did not.

### Data Analysis

rwPFS and OS were measured from date of stage IV or metastatic diagnosis to the date of progression or death, respectively. Progression was defined as a 2 mm increase in primary or metastatic tumor size or treatment change due to clinically determined progression.

### Statistical Analysis

OS and PFS curves were calculated from Kaplan-Meier method.

## Results

### Data Collection

Table 1 describes clinical characteristics and patient demographics including tumor histology and PD-L1 status among the 121 patient cohort. The median age at metastatic diagnosis among patients who continued maintenance pemetrexed was 62 years old, compared with a median of 67 years old for patients who stopped pemetrexed due to toxicity. Among those who stopped pemetrexed due to toxicity, 47% had tumors with PD-L1 less than 1%, compared with 34% of patients in those who did not stop maintenance pemetrexed. Furthermore, 10% of patients who continued maintenance pemetrexed had chronic kidney disease more than or equal to 3, compared with 13% in the group of patients who stopped maintenance pemetrexed due to toxicity. Notably, the median duration of pemetrexed exposure for both groups was 4 months. The median time on total treatment for the group that stopped pemetrexed due to toxicity was 16 months, and the median time on total treatment for the group that continued on pemetrexed was 9.5 months. Of the patients who remained on pemetrexed, 58.8% did not have any subsequent treatments. Furthermore, 30.88% had one line of treatment after pembrolizumab and pemetrexed, 5.88% had two subsequent lines of treatment, and 1.5% had four subsequent lines of treatment. Among patients who stopped pemetrexed due to toxicity, 62.26% had no future lines of treatment, 18.87% had one line of treatment after pembrolizumab and pemetrexed, 9.64% had two future lines of treatment, 1.89% had three subsequent lines of treatment, and 1.89% had four subsequent lines of treatment. Table 2 describes tumor mutations noted in the sample.

**Table 1:** Baseline Demographics of Patients With Nonsquamous Metastatic NSCLC Who Continued on Pemetrexed and Those Who Stopped Pemetrexed

Demographics	Continued Maintenance Pemetrexed (N = 68), n (%)	Stopped Pemetrexed (N = 53), n (%)
Age at Metastatic Diagnosis		
18–64	43 (63)	23 (43)
65–74	20 (30)	20 (38)
75–84	4 (6)	10 (19)
≥85	1 (1)	0 (0)
Sex		
Female	33 (49)	32 (60)
Male	35 (51)	21 (40)
CKD		
≥3	7 (10)	7 (13)
Smoking status		
Current	16 (24)	14 (27)
Former	36 (52)	32 (60)
Never	16 (24)	6 (11)
Unknown	0 (0)	1 (2)
BMI		
≤18.5	2 (3)	2 (4)
18.6–24.9	30 (44)	17 (32)
25.0–29.9	25 (37)	22 (41)
≥30	11 (16)	12 (23)
NSCLC tumor histology		
Adenocarcinoma	61 (90)	48 (90)
Large cell carcinoma	1 (1)	1 (2)
Poorly differentiated carcinoma	5 (8)	3 (6)
Other	0 (0)	1 (2)
No information	1 (1)	0 (0)
Sites of metastases		
Pleural/pleural fluid	17 (25)	8 (15)
Adrenal	14 (21)	6 (11)
Liver	6 (9)	4 (8)
Abdominal/RP LN	1 (1)	0 (0)
Peritoneal/peritoneal fluid	0 (0)	1 (2)
Bone	22 (32)	15 (28)
Brain	17 (25)	16 (30)
Subcutaneous	1 (1)	0 (0)
Diagnosis		
IV at diagnosis	56 (82)	41 (77)
Recurrent, metastatic	12 (18)	12 (23)
ECOG		
0	15 (22)	16 (30)
1	31 (46)	20 (38)
2	7 (10)	5 (9)
Unknown	15 (22)	12 (23)
PD-L1		
<1%	23 (34)	25 (47)
1%–49%	18 (27)	13 (25)
≥50%	7 (10)	7 (13)
Unknown	20 (29)	8 (15)
Line of therapy		
First	64 (94)	51 (96)
Second	3 (5)	2 (4)
Third	1 (1)	0 (0)

BMI, body mass index; CKD, chronic kidney disease; ECOG, Eastern Cooperative Oncology Group.

**Table 2:** Tumor Mutations for Nonsquamous Metastatic NSCLC Who Continued on Pemetrexed and Those Who Stopped Pemetrexed

All Tumor Mutations	Continued Maintenance Pemetrexed (N = 68), n (%)	Stopped Pemetrexed (N = 53), n (%)
Mutations		
EGFR del 19	1 (1)	0 (0)
EGFR E709K,G719A	0 (0)	1 (2)
EGFR exon 20 insertion	0 (0)	1 (2)
EML4-ALK rearrangement	1 (1)	1 (2)
RET rearrangement	0 (0)	2 (4)
MET exon 14 skipping	0 (0)	1 (2)
BRAF non-V600E	2 (3)	0 (0)
BRAF V600E	1 (1)	0 (0)
TP53	29 (43)	24 (45)
STK11	11 (16)	8 (15)
KEAP1	4 (6)	3 (6)
SMARCA4	3 (4)	3 (6)
KRAS G12R	2 (3)	0 (0)
KRAS G12V	8 (12)	2 (4)
KRAS G12C	14 (21)	9 (17)
KRAS G12D	3 (4)	2 (4)
KRAS G12A	3 (4)	4 (8)
KRAS Q61H 183 A>C	0 (0)	2 (4)
KRAS G13D	0 (0)	1 (2)
KRAS G13C	0 (0)	1 (2)

Of reasons listed for pemetrexed cessation, 34% were fatigue, 19% were compounded, cumulative toxicities, and 15% were due to renal dysfunction. Other toxicities included were rash, joint pain, neuropathy, cytopenias, infection, abdominal pain, pneumonitis, colitis, pancreatitis, allergic reaction, endocarditis, and bronchitis.

### Progression-Free Survival

**Figure 1:**
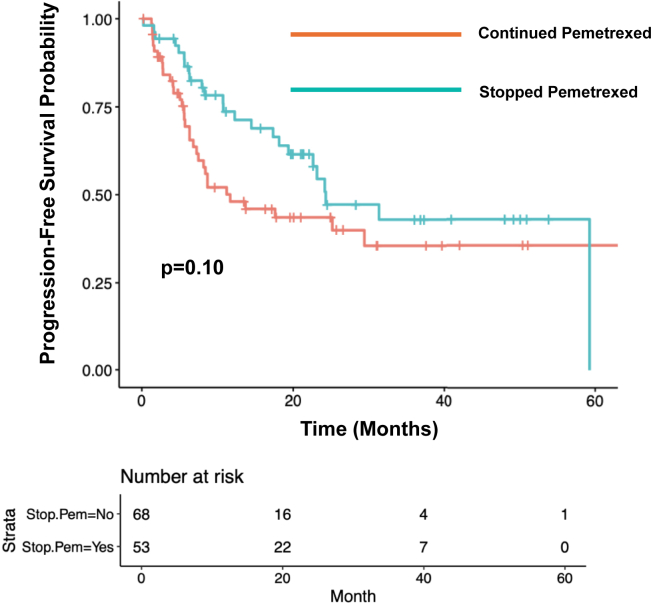
Progression-free survival by decision to stop pemetrexed due to toxicity.

**Figure 2:**
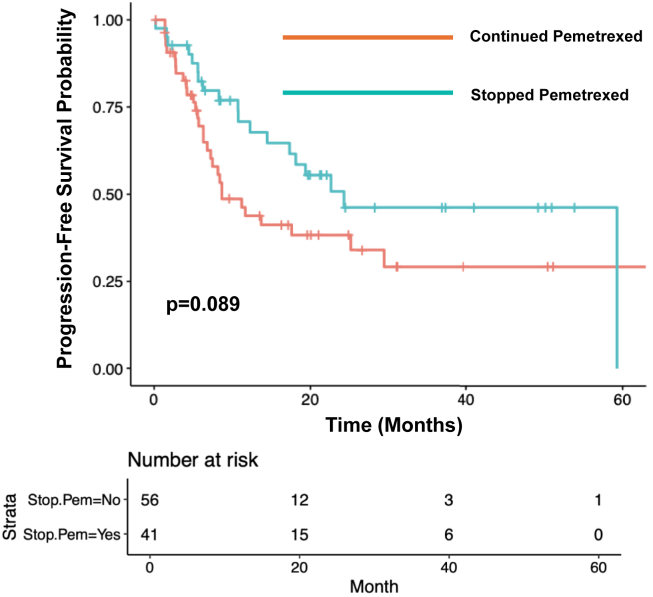
Progression-free survival by decision to stop pemetrexed due to toxicity among patients with stage IV disease at diagnosis.

The median rwPFS among patients who continued maintenance pemetrexed (n = 68) was 11.7 months (95% confidence interval [CI]: 7.47–not applicable [NA]). The median rwPFS among patients who stopped maintenance pemetrexed due to toxicity (n = 53) was 24.3 months (95% CI: 19.37–NA; *p* = 0.10; Fig. 1). Among patients who had stage IV disease at initial diagnosis and who stopped pemetrexed due to toxicity (n = 41), the median rwPFS was 24.3 months (95% CI: 17.33–NA). The median rwPFS among patients with stage IV disease at diagnosis and who did not stop maintenance pemetrexed due to toxicity (n = 56) was 8.67 months (95% CI: 7.23–NA; *p* = 0.09; Fig. 2).

### Overall Survival

**Figure 3:**
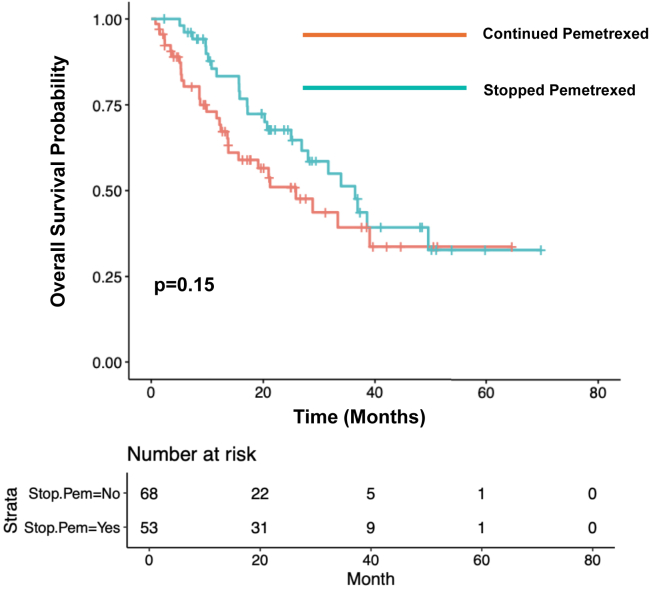
Overall survival by decision to stop pemetrexed due to toxicity.

**Figure 4:**
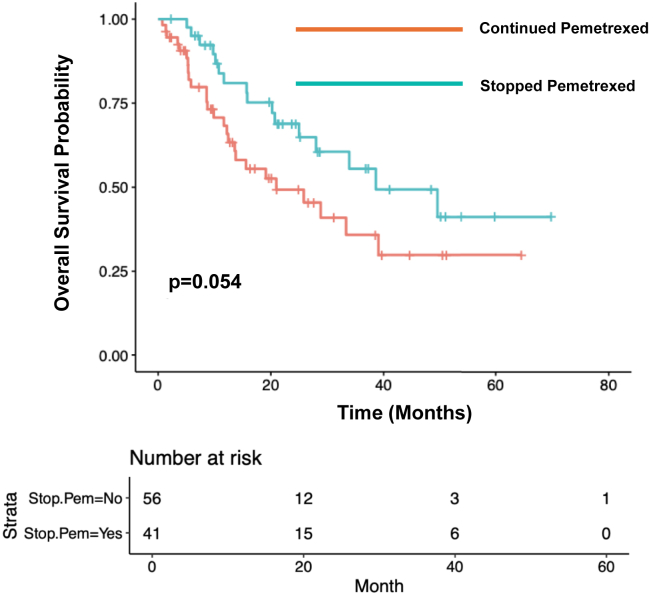
Overall survival by decision to stop pemetrexed due to toxicity among patients with stage IV disease at diagnosis.

The median OS among patients who continued maintenance pemetrexed (n = 68) was 25.8 months (95% CI: 13.8–NA). The median OS among patients who stopped maintenance pemetrexed due to toxicity (n = 53) was 36.4 months (95% CI: 26.9–NA; *p* = 0.15; Fig. 3). Among patients who had stage IV disease at initial diagnosis and who stopped pemetrexed due to toxicity (n = 41), the median OS was 38.6 months (95% CI: 28.0–NA). The median OS among patients with stage IV disease at diagnosis and who did not stop maintenance pemetrexed due to toxicity (n = 56) was 21.0 months (95% CI: 13.6–NA; *p* = 0.05; Fig. 4).

## Discussion

This real-world study provides data to better inform patients with metastatic nonsquamous NSCLC and providers considering cessation of maintenance pemetrexed. Conversations surrounding stopping chemotherapy in chemoimmunotherapy regimens are difficult without clear answers as to how survival may be affected by this change. Our study reveals no significant difference in either rwPFS or OS between patients who stopped pemetrexed due to toxicity versus those who did not. To our knowledge, no previous studies have evaluated the impact of cessation of maintenance pemetrexed on outcomes for patients with NSCLC on chemoimmunotherapy maintenance.

Limitations of this study include its retrospective nature, the single-center data set, and the different thresholds that patients and providers have for tolerability of adverse effects of pemetrexed. Another important limitation of this study was that the median time on pemetrexed was the same for both study groups. The similar rwPFS for both cohorts may reflect that the primary determinant of rwPFS is the underlying individual disease aggressiveness, and a notable cohort of patients who did not stop pemetrexed due to toxicity nevertheless experienced a short rwPFS and limited pemetrexed exposure.

## Conclusion

In conclusion, our data suggest that patients with metastatic, nonsquamous NSCLC placed on maintenance pembrolizumab and pemetrexed who stopped maintenance pemetrexed due to toxicity experience similar clinical outcomes (rwPFS and OS) to patients who continued maintenance pemetrexed. Thus, the optimal duration of pemetrexed maintenance should be reevaluated in the immunotherapy era.

## CRediT Authorship Contribution Statement

**Shreya Bhatia:** Conceptualization, Data Curation, Formal Analysis, Project Administration, Writing - original draft, Writing - review & editing.

**Matthew Lu:** Project administration.

**Spencer Lessans:** Project administration.

**Michael Libre:** Project administration.

**Heidi Chen:** Formal analysis.

**Wade T. Iams:** Conceptualization, Data Curation, Funding acquisition, Investigation, Methodology, Project administration, Resources, Supervision, Validation, Writing - review & editing.

## Disclosure

Dr. Iams has served as a consultant for AstraZeneca, Sanofi, Genentech, Jazz Pharma, G1 Therapeutics, Mirati, Takeda, Janssen, Amgen, Bristol Myers Squibb, OncLive, Clinical Care Options, Chardan, Outcomes Insights, Cello Health, and Curio Science. The remaining authors declare no conflict of interest.
